# The cost of anal cancer in England: retrospective hospital data analysis and Markov model

**DOI:** 10.1186/1471-2458-14-1123

**Published:** 2014-10-31

**Authors:** Sam T Keeping, Michael J Tempest, Stephanie J Stephens, Stuart M Carroll, Karen P Nugent, Sarah T O’Dwyer

**Affiliations:** Sanofi Pasteur MSD, Mallards Reach, Bridge Avenue, Maidenhead, Berks, SL6 1QP UK; Pharmerit Ltd, Enterprise House, Innovation Way, York, YO10 5NQ UK; Faculty of Medicine, University of Southampton, Southampton General Hospital, Mailpoint 801, South Academic Block, Tremona Road, Southampton, SO16 6YD UK; The Christie NHS Foundation Trust, Wilmslow Road, Manchester, M20 4BX UK

**Keywords:** Anal cancer, England, Reimbursement, Resource use

## Abstract

**Background:**

Anal cancer requires a multidisciplinary approach to treatment with often complex interventions. Little is known regarding the associated costs and resource use.

**Methods:**

Patient records were extracted from a national hospital database to estimate the number of patients treated for anal cancer in England. Identified resource use was linked to published UK cost estimates to quantify the reimbursement of treatment through the Payment by Results system. A mathematical model was developed simultaneously to validate findings and to calculate the average 10-year cost of treating a squamous cell anal carcinoma case from diagnosis. The model utilised data from the Association of Coloproctology of Great Britain and Ireland's anal cancer position statement.

**Results:**

On average, 1,564 patients were admitted to hospital and 389 attended an outpatient facility per year. The average annual cost per inpatient and outpatient ranged from £4,562-£5,230 and £1,146-£1,335, respectively. Based on the model estimates, the inflated cost per case was between £16,470-£16,652. Results were most sensitive to the mode of admission for primary treatment and the costs of staging/diagnosis (inflated range: £14,309-£23,264).

**Conclusions:**

Despite limitations in the available data, these results indicate that the cost of treating anal cancer is significant. Further observational work is required in order to verify these findings.

**Electronic supplementary material:**

The online version of this article (doi:10.1186/1471-2458-14-1123) contains supplementary material, which is available to authorized users.

## Background

Anal cancer is a rare disease, accounting for around 4% of large bowel malignancies [1]. More than 80% of the estimated 1,100 cases of anal cancer that are diagnosed each year in the United Kingdom [2] are squamous cell carcinomas, the putative aetiological agent for which is human papillomavirus (HPV) [3], with adenocarcinomas the next most commonly observed tumour (~10%) [1]. Far less common are anal melanomas, lymphomas and sarcomas [1]. Some evidence suggests that the incidence of anal cancer is increasing, with age standardised rates per 100,000 rising from 0.7 to 1.1 and 0.6 to 1.3 between 1986 and 2003 in English males and females, respectively [4, 5]. Recent epidemiology studies have postulated that the increase in anal cancer incidence is attributable to changes in sexual behaviour (i.e. a higher number of unprotected receptive anal sex partners), a likely surrogate for infection with multiple high-risk HPV strains [6]. Interestingly the incidence of anal cancer has dramatically increased among HIV-infected men, despite antiviral therapy (e.g. during and proceeding the HAART era) [7]. One potential explanation for this is that whilst antiviral therapy may reduce competing mortality risks, it has no impact on the impact on the natural history of HPV nor the likelihood or HPV co-infection and, moreover, increasing life expectancy allows sufficient time for the accumulation of genetic mutations implicated in the development of anal cancer. Such data highlights the need for preventative strategies for anal cancer [7].

Anal cancer is a slow progressing disease and local disease failures after primary treatment normally go on to develop metastatic disease. Therefore, the main goal of curative treatment is achieving adequate local control [8]. Optimal primary treatment involves chemoradiotherapy, although a small number of patients are unable to tolerate the full treatment regime and are at risk of residual disease as a result [9]. Radical salvage surgery remains the primary option for those who experience locoregional relapse [10].

Changes in the treatment approach away from primary surgical intervention have required a shift to a multi-disciplinary model of patient management [1]. In England and Wales, specialist anal cancer multi-disciplinary teams (MDTs) have been established within and across cancer networks, with all referrals of suspected cases being discussed during regular meetings [11]. This is also in keeping with a general strategy aimed at improving outcomes for rarer cancers, with similar arrangements in place for penile cancer, which is another HPV-related genital cancer [12].

In contrast to the well-developed literature on *treatment* options for anal cancer, very little has been published on the costs and resource use associated with treating the condition. In the last ten years, only two economic evaluations of treatment for anal cancer have been carried out in the UK setting [4, 13]. Both of these extrapolated costs from research on other cancers: one from a study of colorectal cancer [14] and the other from two studies of cervical cancer costs [15, 16].

In order to inform future economic analysis, this study reports an estimate of both the mean annual costs of treating anal cancer in England, and also the average cost of treating a single case of the most common type of anal cancer, squamous cell carcinoma, in line with the Association of Coloproctology of Great Britain and Ireland's anal cancer position statement [1, 8, 11, 17–21].

## Methods

The study was split into two phases. Firstly, a retrospective (non-comparative) case series was performed using data extracted from the Hospital Episode Statistics (HES) database. HES includes records of all care funded by the English National Health Service (NHS), allowing the economic burden associated with pre-cancerous and invasive anal cancer lesions in England to be estimated. Due to the short span of extracted data years, the exclusion of primary care, the inability to distinguish between initial and recurrent cases, and the lack of information pertaining to the cancer stage; a separate mathematical model was developed to simulate the treatment pathway for an anal cancer case of squamous origin in order to estimate the average cost of treating a single patient.

### HES data collection

For inpatients, finished consultant episodes (FCE) were extracted based on the presence of any of the following International Classification of Diseases, 10^th^ (ICD-10) codes in either the primary, secondary of tertiary diagnosis field: C210 – malignant neoplasm of anus, unspecified; C211 – malignant neoplasm of anal canal, C212 – malignant neoplasm of cloacogenic zone, C218 - malignant neoplasm overlapping lesion of rectum, anus and anal cancer, and D013 – carcinoma in situ of anus and anal canal. Data on outpatient attendances were confined to records with a primary or secondary diagnosis only, reflecting the more disease specific nature of post-treatment care.

Data were collected for care delivered in the period from 2006 to 2011. As our HES data request was submitted prior to the publication of full year results for 2010/2011, the final year was only representative of nine months of activity in both patient settings.

Approval for use of the HES data was provided by the information asset owner from the Health and Social Care Information Centre (HSCIC). The individual HES records extracted contained no sensitive data and were pseudonymised preventing the true identification of patients; analyses pertaining to HES records adhered to published regulations [22]. Ethical approval was not required as secondary analysis of HES data can be used to identify public health issues and for general medical research under existing protocol; the Health Research Authority decision tool corroborated this fact stating no ethical approval was required for this research [23].

### Data aggregation and costing

The number of patients undergoing treatment for anal cancer in each year of the study period was determined by tracing the unique patient identifiers (HESID) assigned to each FCE. Mean annual patient numbers were then calculated. HES data aggregation and the generation of descriptive statistics was carried out using SAS Enterprise Guide 4.3.

NHS funded healthcare providers in England are reimbursed under the Payment by Results (PbR) scheme [24]. The currencies for payment under PbR are healthcare resource groups (HRG). Each year, payment tariffs for each HRG are determined using retrospective analysis of costing data submitted from previous years. In order to derive relevant HRGs for care delivered to anal cancer patients, inpatient FCEs were aggregated into spells of care (from hospital admission to discharge) using software publicly available from the NHS [25]. Similarly, for non-admitted patient care (outpatient), Treatment Function Codes (TFC) and associated HRGs for consultations or minor procedures were generated. For all core HRGs and TFCs, costs were calculated using the National Tariff 2010/11 [26]. Costing analyses were performed in Excel 2007.

Costs were applied on a per spell basis, accounting for adjustments such as those for non-elective admissions, and then aggregated by year. For the final year in the study period, for which data was only representative of nine months of activity, costs and patient numbers were scaled up by a factor of 1.33, assuming no seasonality in treatment patterns or propensity to consult.

The annual estimates were then used to calculate mean annual costs for the duration of the study period, with mean annual cost per patient calculated by dividing this by the mean annual number of patients. All analyses were split by gender, with results for those diagnosed with carcinoma in situ of the anus and anal canal also presented separately to those with invasive disease due to the anticipated differences in treatment intensity for this group.

Under the latest version of HRGs (version 4), some significant elements of cost and activity (e.g. chemotherapy, radiotherapy and rehabilitation) have been ‘unbundled’ from the core HRGs. The frequency of unbundled HRGs was also captured, with costs associated with the procurement and administration of chemotherapy and radiotherapy also disaggregated. Unbundled HRGs are excluded from the National Tariff due to wide variation in regional practices and costs, with prices negotiated locally. To derive costs for these unbundled HRGs, the associated codes were cross-referenced and matched by description to codes in the Reference Cost Tariffs [27] for the year of interest. Earlier tariff years were utilised in the presence of unmatchable codes and inflated accordingly using the consumer price index (CPI) [28].

### Estimating costs over the duration of treatment

**Figure 1:**
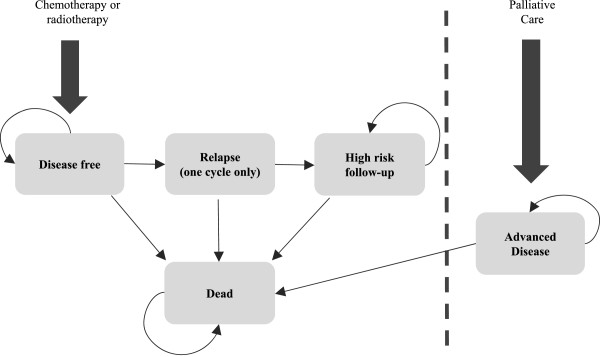
**Markov model structure.**

To estimate the average cost of treatment for a case of anal cancer a mathematical model was constructed to capture the costs of referral through to follow-up, taking into account the impact of primary treatment on the duration and intensity of the latter. Due to a lack of data on outcomes for patients with primary adenocarcinoma, the model was restricted to anal cancer patients with squamous cell carcinoma. Data on primary treatment, disease progression and follow-up for this group were obtained from the Association of Coloproctology of Great Britain and Ireland's anal cancer position statement, supplemented by expert opinion where necessary. First, decision trees were constructed to estimate the costs of diagnosis, staging, including pre-treatment interventions such as colostomy surgery, and primary treatment. A Markov model was then developed to simulate disease progression and follow-up based on the mode of primary treatment (chemo radiotherapy vs. radiotherapy).The structure of the Markov portion of the model can be seen in Figure 1. A one month cycle length was used and the model was run over a timeframe of ten years (the extent of high risk follow-up). The initial conditions assumed that all patients who receive either radiotherapy or chemo radiotherapy as primary curative treatment are left disease free. Patients experiencing relapse were assumed to stay in the relapse state for one month only, representing the period during which salvage interventions occur. Salvage was assumed to be in the form of abdominoperineal resection (APR).

Monthly probabilities for relapse, death from the disease free-state and death post relapse (assumed to be the same for those undergoing salvage or follow-up) were estimated by calibrating the model to empirical data. Values for these unknown parameters were jointly selected from within plausible ranges, with simulations run in batches of 1,000. The best fitting set of values, determined by comparing the model output to longitudinal data on overall mortality and loco-regional relapse [29] using least squares from each batch of simulations were then used to determine the ranges from which values were selected in the next round. We ceased to run simulations at the point when no improvements were observed in the overall goodness-of-fit at three significant figures.

All costs applied to treatments and interventions were taken from the 2010/11 National Tariff, with the 2010/11 Reference Costs used for off tariff payments. Two scenarios for pricing were examined: 1) with no future inflation in prices and 2) where future inflation was assumed to remain constant at 2011/12 levels for the duration of follow-up [28]. The aim of our approach was to estimate realised costs as opposed to modelling an investment decision, therefore discounting was deemed to be inappropriate. A one-way sensitivity analysis was also performed for the following key inputs: type of admission for primary treatment, cost of staging and primary treatment, monthly costs of palliative care, intensity of follow-up, proportion of patients undergoing salvage surgery, proportion of patients presenting with advanced disease, cost of follow-up interventions and the proportion of patients receiving chemo radiotherapy.

Full details of all the model inputs can be found in the Additional file 1.

## Results

### Number of patients

From 2006-2011, the mean number of patients admitted to hospital each year in England for invasive anal cancers was 1,564 (Table 1). Approximately 30% more females than males were admitted each year on average (mean: male = 642; female = 922). Over the same period on average 389 patients attended outpatient facilities each year for anal cancer (mean: male = 139; female = 250). The mean start age for anal cancer treatment was 64 and 65 for males and females, respectively (data not shown).

**Table 1:** **Mean annual number of patients, hospital spells, outpatient attendances and cost per patient (2006-2011)**

	Inpatient			Outpatient		
	Number of spells (SD)	Number of patients (SD)	Cost per patient (SD)	Number of attendances (SD)	Number of patients (SD)	Cost per patient (SD)
**Anal cancer**						
Male	1743 (69)	642 (87)	£4,562 (£431)	1377 (381)	139 (21)	£1335 (£596)
Female	2931 (290)	922 (81)	£5230 (£332)	2187 (727)	250 (65)	£1146 (£374)
**Anal carcinoma in situ**						
Male	70 (5)	58 (14)	£881 (£336)	8 (16)	1 (1)	£1240 (£1276)
Female	133 (32)	110 (32)	£1145 (£126)	7 (2)	2 (1)	£326 (£70)

SD, Standard Deviation.

### Hospital spells and outpatient attendances

On average, there were 4,674 hospital spells and 3,564 outpatient attendances each year. Male patients were associated with a mean of 3 hospital spells and 10 outpatient attendances. Female patients had the same number of hospital spells on average but one less outpatient attendance. Across genders, the mean length of an inpatient hospital stay was 4 days.

Eighty-five per cent of hospital spells were elective admissions and 33% were day case admissions across both genders. Excess bed days were observed in 10% of male elective hospital spells and 11% of female elective hospital spells. For non-elective spells, excess bed days were observed in 3% for both males and females.

The most frequent HRGs were diagnosis driven anal disorders of varying severity, representing 47% of male hospital spells and 51% of female hospital spells. Anal procedures were the second most observed HRG across both genders accounting for 8% of hospital spells. Large intestine disorders and procedures were observed in 12% and 8% of male and female hospital spells, respectively.

Chemotherapy sessions (procurement and delivery) were observed in 12% and 11% of male and female hospital spells. Radiotherapy sessions (procurement and delivery) were observed in 7% of male hospital spells and 9% of female spells. Across genders, palliative care was associated with 1% and rehabilitation <1% of hospital spells.

Minor outpatient procedures were observed in 2% of all attendances. Follow-up consultations were the most frequent HRG observed in 90% of male and 89% of female attendances. The most frequent TFC observed was clinical oncology in 87% and 89% of male and female attendances, respectively. Chemotherapy sessions were observed in 1% of male and <1% female attendances, radiotherapy sessions were observed in 29% and 33% of male and female attendances, respectively. Palliative care and rehabilitation was observed in <1% of all attendances.

### Economic burden

All costs presented considered a direct healthcare perspective and are presented in GBP (2011 prices). The mean annual cost per patient (setting dependent) is provided in Table 1. The mean annual cost per inpatient was estimated at £4,562 and £5,230 for males and females, respectively. Costs per outpatient were calculated at £1,335 for males and £1,146 for females.

Table 2 provides the total annual cost for anal cancer defined by gender and per type of care.

**Table 2:** **Mean total annual costs per category of cost from the payers’ perspective**

	Inpatient				Outpatient				Total
	Bundled (SD)	Unbundled* (SD)	Chemo-therapy (SD)	Radio-therapy (SD)	Bundled (SD)	Unbundled* (SD)	Chemo-therapy (SD)	Radio-therapy (SD)	
**Anal cancer**									
Male	£2,658,450 (£152,591)	£61,026 (£46,809)	£88,783 (£36,680)	£122,103 (£34,585)	£120,265 (£32,687)	£5,096 (£7,034)	£8,332 (£12,664)	£51,214 (£57,976)	£3,115,267
Female	£4,351,222 (£338,353)	£88,273 (£49,798)	£132,493 (£52,957)	£251,870 (£50,504)	£191,725 (£62,773)	£6,426 (£8,774)	£2,420 (£3,082)	£86,129 (£86,632)	£5,110,560
**Anal carcinoma in situ**									
Male	£49,272 (£8,042)	£563 (£1,196)	£487 (£614)	£1,144 (£1,527)	£744 (£1,276)	£0	£0	£0	£52,211
Female	£125,091 (£32,803)	£133 (£209)	£296 (£446)	£407 (£772)	£553 (£186)	£141 (£216)	£0	£0	£126,622

*Excludes chemotherapy and radiotherapy; SD, Standard Deviation.

For male inpatients, £2,658,450 was attributable to bundled costs which include all care received in a hospital setting excluding unbundled HRGs such as chemotherapy, radiotherapy, rehabilitation, palliative care, specific diagnostic imaging and high cost drugs. Annual unbundled costs (excluding chemotherapy and radiotherapy) were equal to £61,026. Respectively, chemotherapy and radiotherapy cost £88,783 and £122,103 per year.

Of the total burden associated with male outpatients, £120,265 was attributable to bundled costs and £5,096 to unbundled elements of care (excluding chemotherapy and radiotherapy). Respectively, chemotherapy and radiotherapy cost £8,332 and £51,214 per year.

**Figure 2:**
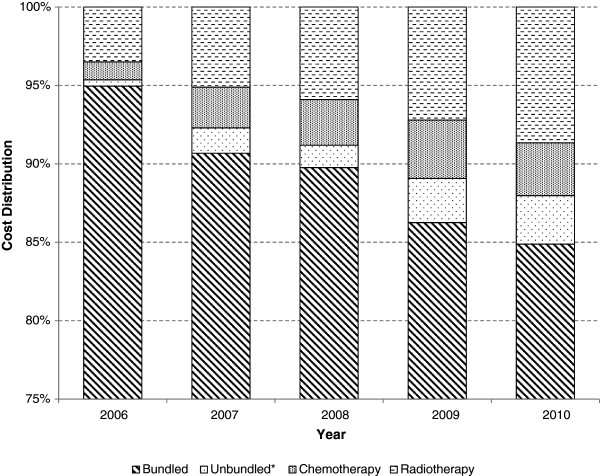
**Total annual cost distribution per category of care for invasive anal cancer.** * Excludes chemotherapy & radiotherapy.

Female anal cancer patients were associated with payments for bundled HRGs totalling £4,351,222 for inpatients and £191,725 for outpatients; £88,273 for inpatient and £6,426 outpatient unbundled costs (excluding chemotherapy and radiotherapy); £132,493 and £2,420 for inpatient and outpatient chemotherapy, and £251,870 and £86,129 for inpatient and outpatient radiotherapy, respectively.Across genders and settings, bundled costs accounted for 89% of the total annual burden. Unbundled costs accounted for 2%, chemotherapy and radiotherapy accounted for 3% and 6%, respectively. Analysing this trend, it is clear that the proportion of the cost attributable to chemotherapy and radiotherapy has been increasing, although this is thought to be predominantly due to improvements in correct clinical coding as opposed to changes in the treatment paradigm (Figure 2).

### Total costs for duration of treatment

**Figure 3:**
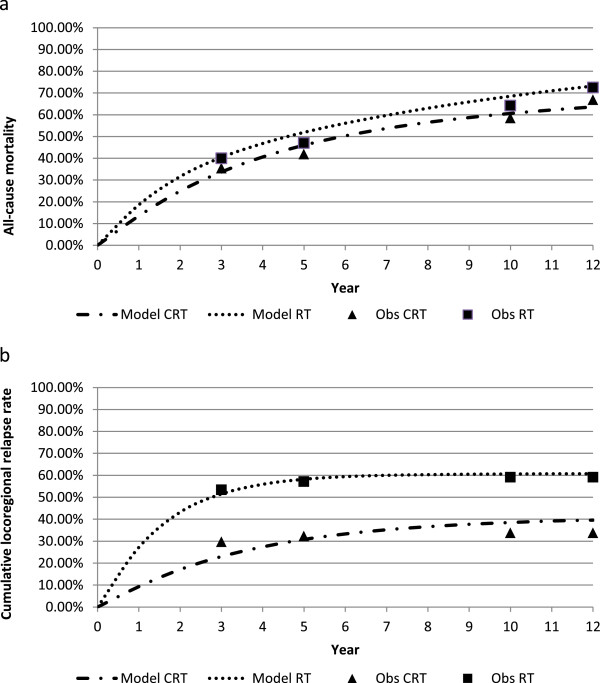
**Predicted a) all-cause mortality and b) cumulative relapse compared to data from the UKCCCR anal cancer trial (ACT 1).**

Figure 3 shows the model predictions for cumulative all-cause mortality and relapse, compared to data from the UKCCR Anal Cancer Trial (ACT 1), for the best fitting set of parameters which could be achieved through the calibration process.

The average cost of treating a case of invasive anal cancer from referral through to either completion of follow-up or death was estimated to be £16,473 (between £14,335 and £23,089 based on sensitivity analysis) when future inflation was taken into account and £16,281(between £14,143 and £22,884) when it was not. For those undergoing treatment with curative intent, the majority of costs were accrued during staging, primary treatment and the twelve months of follow-up post referral, with annual costs in the following years ranging from £338 to £815 (uninflated £308-£807) and £363 to £1,452 (uninflated £338-£1,437), for those undergoing chemoradiotherapy and radiotherapy alone, respectively.

**Figure 4:**
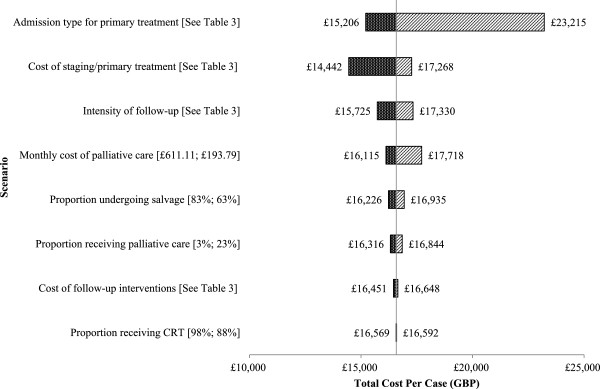
**Tornado diagram detailing the one way sensitivity analysis.**

Figure 4 shows the results of the one-way sensitivity analysis used to construct the ranges around the base case estimates for the inflated scenario (Table 3). The results were most sensitive to changes in the mode of admission for primary treatment, reflecting the higher costs associated with primary treatment that requires longer patient stays. Changes in the cost of staging and primary treatment also had a marked impact on the results, highlighting once again that initial interventions were the main drivers of total costs. Another implication of this was that the proportion undergoing salvage had a limited impact on the results, despite these procedures being extremely resource intensive.

**Table 3:** **Upper and Lower values implemented in the one-way sensitivity analysis**

Scenario/variable	Upper value	Lower value
Admission type for primary treatment*		
Radiotherapy planning	£2,869	£729
PICC line insertion	£2,068	£751
Procure 5-FU	£339	£286
Initial 5-FU cycle delivery	£334	£302
Subsequent 5-FU cycle delivery	£294	£206
Radiotherapy delivery	£261	£111
**Cost of staging/primary treatment** ^**†**^		
MRI scans	£413	£235
CT scans	£177	£116
Fine needle aspiration	£616	£269
Examination under anaesthetic/biopsy	£1,797	£1,117
Radiotherapy planning	£1,309	£674
Mytomycin C	£48	£40
PICC line insertion	£877	£414
Procure 5-FU	£360	£187
Initial 5-FU cycle delivery	£401	£232
Subsequent 5-FU cycle delivery	£343	£210
Radiotherapy delivery	£134	£122
**Intensity of follow-up**		
*Low-risk of relapse*		
Follow-up appointments	18	14
MRI scans	2	0
CT scans	2	0
*High-risk of relapse*		
Follow-up appointments	18	14
MRI scans	7	5
CT scans	4	2
**Cost of follow-up interventions**		
MRI scans	£413	£235
CT scans	£177	£116

*Upper and lower values are for inpatient and outpatient costs, respectively.

^†^Based on upper and lower quartile of the reference costs for each item.

## Discussion

With incidence of anal cancer on the increase, better information on treatment costs will assist in defining future service specifications for commissioning and delivery of treatment. Although not an incremental analysis in itself, this study provides the first attempt to estimate the cost of treating anal cancer in England, and potentially allows for better assessment of the cost-effectiveness of new treatments for anal cancer through the determination of the costs of current best available treatment. As with any new data analysis, the research suffers from some limitations. These mainly stem from the structure of the HES database and the lack of available data on disease progression, and associated treatment patterns, for anal cancer.

Firstly it should be acknowledged that despite the non-comparative case-series study design, the inherent bias noted with this approach was limited in the present study. Patient records were extracted from all English hospitals based on the aforementioned ICD-10 codes only with no additional inclusion or exclusion criteria applied. Furthermore, cases were retrieved retrospectively with all information collected routinely in relevant medical records. Nonetheless, restricting the presence of an ICD-10 code up to the tertiary and secondary diagnoses fields for inpatient and outpatients, respectively may have introduced some selection bias, for example by excluding adverse treatment events such as neutropenic sepsis and radiation proctitis. However, relaxing the this restriction may have introduced costs completely unrelated to anal cancer.

In the HES portion of the analysis, it is evident that the link between the MDT and clinical coders is inefficient; most notably in the earlier data years extracted. Fields such as patient age, sex, admission and discharge methods, hospital provider codes and codes pertaining to diagnosis and operational procedures are mandatory in the grouping process from FCEs to spells of care and the derivation of the dominant and correct HRGs; the prevalence of missing fields and thus erroneous coding was higher within these data years, leading to potential underestimations within the economic analysis. The coding errors are recognised under PbR and frameworks are in place to reduce the percentage of errors in order to improve the quality of data that underpins PbR. Between 2007/08 and 2009/10, the audit commission looked at over £200 million of payments for admitted patient care, and observed an improvement in the accuracy of clinical coding during this period. The coding error rate dropped from 16% to 11% in three years, although there remains a wide variation between the best and worst performing providers. Coding errors occur at a clinical level and ultimately affect payments. The Audit Commission estimate that of the £21 billion spent on the four specialities they audited for three years, £1 billion (5%) was incorrectly paid [24].

Furthermore, despite HES records containing exhaustive data fields, other key information pertaining to a patient’s diagnosis would have proved valuable within the analyses. For example, ICD-10 codes provide a platform for extracting all records related to anal cancer. However, it does not provide information pertaining to a patient’s cancer stage, or if a tumour is primary vs. recurrent. One would naturally assume a higher cost and more intense resource use as the cancer stage progresses. This distinction could not be made and therefore restricted the performed analysis.

The length of time over which resource use could be analysed was also constrained by changes to the coding methodology used in PbR. Data was originally extracted from 2002/03 to 2010/11 and spanned several versions of HRG. When aggregating FCEs into spells of care, it was noted that data prior to 2006 omitted chemotherapy and radiotherapy unbundled HRGs. The reason for this was traced to the HRG4 grouping software requirements on OPCS 4.3 codes for procurement and delivery, which were not authored and released to the NHS until April 2006. This resulted in severe data gaps associated with unbundled HRGs from 2002/03 to 2005/06 hence the decision was taken to restrict the analysis to data from 2006 to 2011.

It should also be noted that outpatient data prior to 2006 was deemed as experimental data due to the immaturity of the clinical coding process in this setting. Data after this year are accredited as a national statistic. However, it was increasingly apparent from analysis and observations of the dataset that this is not of equal quality to the inpatient dataset. Clinical experts noted that the observed outpatient numbers were lower than expected: it is expected a patient admitted to hospital would attend the outpatient facility at least once, which was not the case here.

High cost drugs are excluded under PbR and no indication of hospital prescribing data is available within HES. The exclusion of such costs is acknowledged as an analytical limitation. Furthermore, costs associated with unbundled HRGs are not available in the National Tariff 2010/11 due to wide regional variations in resource use and cost; such prices are locally negotiated. We therefore used the national average cost from the National Reference Costs, which provides the foundations for the National Tariff. Definitions for HRGs can alter yearly and inaccurately matching codes and definitions could have potentially led to an underestimation or overestimation.

Nonetheless, the HES analysis does provide the most complete analysis of the overall burden of anal cancers to date and is based on a national database currently used for a range of healthcare analyses for the NHS and Government. It is from this perspective that HES can be seen as a useful starting point for costing analyses. These estimates show that there is a significant burden associated with treatment of the various tumours and provide a benchmark against which any future efficiency improving measures can be judged. Our research also highlights the limitations of the HES database and difficulty of calculating the overall burden of specific cancers in England. Initiatives to improve clinical coding and the functioning of PbR will be important in aiding future efforts to quantify the cancer burden.

The mathematical modelling was subject to inherent data limitations. Firstly, due to a lack of data on stage at diagnosis and treatment outcomes, we had to assume that for all patients treated with curative intent, regardless of stage, local control was achieved. Although this assumption is unrealistic, its impact is limited as the model allows patients to relapse from the first month post-treatment (assuming a two month gap between treatment and assessment for response) therefore mirroring the treatment which would likely be provided to those with residual disease. Secondly, data on palliative care was also limited requiring assumptions about treatment based on expert opinion, with monthly costs extrapolated from studies of resource use patterns for other tumours. Finally, costs for care delivered during follow-up outside of the standard pattern of appointments, for example those related to stoma nurses, could not be included in the model due to a lack of available information.

The memory-less property of the Markov model also meant that it was not possible to determine when a particular patient had entered the post-relapse state, and consequently it was not possible to accurately apply the costs of interventions delivered as part of the specified programme of follow-up for high risk patients. Monthly follow-up costs post-relapse were therefore based on an average monthly cost associated with a completed programme of interventions for those with a high-risk of relapse. This inability to account for the distribution of costs for follow-up post salvage surgery, and also the likely need for palliative interventions, has probably led to some overall underestimation in the total costs.

It was also assumed that patients receiving salvage surgery would also undergo some cosmetic or reconstructive surgery. The impact of this assumption was limited by the small amount that salvage surgery contributed to total costs overall. It was also not possible to capture the long term follow-up costs of those undergoing pre-treatment colostomy, 90% of which are not reversed. Overall, the assumptions used suggest that the model is very conservative in estimating overall treatment costs.

Despite these data limitations, the model achieved a good fit to published data on the natural history of anal cancer from follow-up data on participants in ACT 1. The inclusive nature of ACT 1 (those with anal margin and T1 tumours were not excluded despite their favourable prognosis) also helps with the generalisability of the results. Although the model results are not entirely congruent with the per patient costs observed in HES, this is posited to be the result of annual increases in new patients pushing per patient costs upwards due to more resource intensive treatment. The mean annual costs from the model are also deflated somewhat by the ten year timeframe used. The inclusion of tumours other than those of a squamous nature, which are the focus of the mathematical model, may also have contributed to the observed differences.

These data are timely given the increasing number of new interventions aimed at anal cancer, including monoclonal antibodies which have been used to treat other squamous cell carcinomas, such as cetuximab (ERBITUX; ImClone Systems Inc., New York, NY, and Bristol-Myers Squibb Co, Princeton, NJ), and vaccines against HPV (e.g. Gardasil, a quadrivalent vaccine developed and marketed in the UK by Sanofi Pasteur MSD). Sufficient evidence is available to support the causal relationship between HPV-infection and malignant growths of the anus [30–35]. Low-risk HPV subtypes are more commonly associated with low-grade dysplasia, in contrast to high-risk HPV subtypes (e.g. HPV-16, 18), which are more frequently identified in carcinoma in situ or invasive carcinoma lesions. Population based epidemiological studies suggest between 63% and 90% of invasive anal cancers are attributable to HPV [30–35].HPV-16 is the most frequently detected HPV subtype and is prevalent in an estimated 48.6% to 76.5% of anal cancer cases [30, 31, 33, 35]. Hitherto, economic evaluations of the cost-effectiveness of HPV vaccination in the UK have had to use anal cancer treatment costs extrapolated from other sources. Our findings can be used as an alternative and contribute to a more accurate assessment of the potential value of HPV vaccination.

## Conclusions

Despite being a rare condition, anal cancer patients place a significant burden on NHS resources in England. It is expected that this should decrease as the impact of the current HPV vaccination programme for adolescent girls takes effect. Nonetheless, the reduction may be less than expected given the group most affected by anal cancer, men-who-have-sex-with-men (MSM), do not benefit from the herd protection afforded by the girls programme. Our estimated treatment costs could now be used to better estimate the value and cost-effectiveness of vaccination programmes, particularly those targeted at the male and MSM populations. This could have important implications in terms of the expected net health benefit resulting from preventative healthcare interventions versus existing treatment options and in turn the potential cost offsets to the NHS. The research also provides a benchmark against which further changes to the overall structure of service delivery for anal cancer patients can be judged.

## Electronic supplementary material

Additional file 1: **Model description and parameters.** (DOC 372 KB)

## Abbreviations

ACT1: Anal cancer trial 1
APR: Abdomino perineal resection
CPI: Consumer price index
FCE: Finished consultant episode
GBP: Great british pounds
HES: Hospital episode statistics
HESID: Hospital episode statistics patient identifier
HPV: Human papillomavirus
HRG: Healthcare resource group
ICD-10: 10th revision of the international statistical classification of diseases and related health problems
MDT: Multi-disciplinary team
MSM: Men-who-have-sex-with-men
NHS: National health service
NICE: National institute of health and clinical excellence
OPCS: Office of population censuses and surveys classification of interventions and procedures
PbR: Payment by results
TFC: Treatment function code
UK: United Kingdom
UKCCR: United Kingdom coordinating committee on cancer research.
