# Supplementary material for: The cost of anal cancer in England: retrospective hospital data analysis and Markov model
Source: BMC Public Health. 2014 Oct 31;14:1123. doi: 10.1186/1471-2458-14-1123 (PMC4232734; doi:10.1186/1471-2458-14-1123)
Supplement: Supplementary file 1 — Additional file 1: Model description and parameters. (DOC 372 KB) [file 12889_2014_7225_MOESM1_ESM.doc]

**The Cost of Anal Cancer in England**

**Additional file 1: Online Material**

All the inputs used in the mathematical model portion of the analysis are detailed below. Each section also includes a brief discussion of specific inputs and any assumptions used in their selection.

1. Referral, Staging and Primary Treatment Probabilities
   1. Referral

Table 1: Source of referral probabilities

| Parameter |  | Value | Reference |
| --- | --- | --- | --- |
| GP |  | 0.850 | Expert discussion |
| GUM physician |  | 0.150 |  |

No data exists on referral routes for patients with suspected anal cancers. Assumed values were based on the expectation that older patients, who make up the vast majority of the overall burden, would be referred by their General Practitioner (GP). In contrast, cases in younger individuals, particularly men-who-have-sex-with-men, would be more likely to be referred by a physician at a genitourinary medicine (GUM) clinic.

- 1. Staging

Table 2: Probability of different interventions during staging process

| Parameter |  | Value | Reference |
| --- | --- | --- | --- |
| Initial consultation  Biopsy  MRI scan  CT scan  Node sampling (fine needle aspiration)  Pre-treatment colostomy |  | 1.000  1.000  1.000  1.000  0.250  0.058 | Expert discussion  Expert discussion  Expert discussion  Expert discussion  [1]  [2] |

In the base case scenario, all patients were assumed to receive MRI and CT scans as well as undergo a biopsy of the primary lesion [1]. A minority of cases also underwent node sampling by fine needle aspiration. For those with advanced disease at diagnosis, pre-treatment colostomy is often necessary. The value used for this variable was based on audit data from the Greater Manchester and Cheshire Cancer Network (GMCCN) which covers a population on 3.2 million people [2].

- 1. Primary Treatment

Table 3: Probability of treatment intent

| Parameter |  | Value | Reference |
| --- | --- | --- | --- |
| Curative |  | 0.867 | [2] |
| Palliative |  | 0.133 | [2] |

The split between curative and palliative treatment was determined from data on new registrations of anal cancers of a squamous nature by the GMCCN between 2004 and 2009 [2].

- - 1. Curative Treatment

Table 4: Primary curative treatment probabilities

| Parameter |  | Value | Reference |
| --- | --- | --- | --- |
| Chemoradiotherapy |  | 0.926 | [2] |
|  | Booster | 0.913 | [3] |
| Radiotherapy |  | 0.074 | [2] |
|  | Booster | 0.843 | [3] |

All patients undergoing treatment with curative intent were assumed to receive either a combination of chemotherapy and radiotherapy or radiotherapy alone. Some patients do undergo primary surgical interventions and accounting for these within the model was explored. However, the small numbers combined with a lack of data on long term outcomes for the different surgical modalities led us to the view that their inclusion would be unlikely to materially affect the final results but would introduce significant additional uncertainty to the model estimates. Therefore, we opted to retain the treatment groupings based on whether patients were or weren't able to tolerate concomitant chemotherapy during radiotherapy.

The proportion of patients unable to tolerate chemotherapy was also taken from the GMCCN audit data [2], with proportions completing the full course of radiotherapy i.e. receiving booster fractions as part of the recommended treatment regime taken from a retrospective study of patients treated in the same network between 1988 and 2000 [3].

- - 1. Palliative Treatment

Table 5: Palliative treatment probabilities

| Parameter |  | Value | Reference |
| --- | --- | --- | --- |
| Radiotherapy |  | 0.500 | Expert discussion |
| Surgery |  | 0.500 |  |

Data on palliative care for anal cancer patients is very limited. After expert discussions it was agreed that it was reasonable to assume some form of intervention would be administered prior to standard supportive care. We therefore assumed an even split between minor surgical and radiation treatment.

1. Markov Model

In order to simulate the impact of the different primary treatment options on the intensity of follow-up, a Markov model was constructed.

- 1. Model Structure

The model structure can be seen in Figure 1 (see manuscript). The model is split in two, with the distinction based on whether patients are treated with curative or palliative intent.

The model was further separated in the curative component into those receiving combination therapy and those receiving radiotherapy alone. It was assumed that local control was achieved in all patients undergoing combination therapy or radiotherapy alone with curative intent regardless of whether the full course was completed or not. Although this assumption is unrealistic, it's impact is limited as the model allows patients to relapse from the first month post treatment, therefore mirroring the treatment which would likely be provided to those with residual disease.

From the disease free state, patients could either stay disease free, experience locoregional relapse or die. For costing purposes, a proportion of those moving into the relapse state during each cycle were assumed to undergo salvage surgery. Patients were only able to spend one cycle in the relapse state after which they either moved to the post-relapse (high risk follow-up) state or died. No additional mortality was assumed for the initial relapse state versus that in the post-relapse state due to the lack of distinction between those undergoing salvage and those who do not. This approach was taken for two reasons. Firstly, the aim was to keep the model as simple as possible in order to minimise the uncertainty in the estimates. Secondly, this approach represented the conservative option as treating all patients with relapse as a homogenous group was associated with lower survival, hence lower costs of follow-up.

Patients undergoing palliative care could either continue to receive care or die.

- 1. Calibration

All transition probabilities were calculated by assuming an exponential relationship between time and the events in question. Values for unknown parameters were jointly selected from within plausible ranges, with simulations run in batches of 1,000. The initial ranges used in the calibration process can be found in the following sections.

The best fitting set of values, determined by comparing the model output to longitudinal data on overall mortality and locoregional relapse [4] using ordinary least squares, from each batch of simulations were then used to determine the ranges from which values were selected in the next round. We ceased to run simulations at the point when no improvements were observed in the overall goodness of fit at three decimal places.

- - 1. Initial Ranges Curative Chemoradiotherapy

Table 6: Initial ranges for transition probabilities associated with curative chemoradiotherapy used in calibration process

| Variable |  | Range | Reference |
| --- | --- | --- | --- |
| Disease-free → disease-free | | 0.985-0,992 | [4] |
| Disease-free → locoregional relapse | | 0.002-0.004 | [4] |
| Disease-free → death | | 0.006-0.011 | [4] |
| Locoregional relapse → post-relapse follow-up | | 0.973-0,984 | [3] |
| Locoregional relapse → death | | 0.016-0.027 | [3] |
| Post-relapse follow-up → post-relapse follow-up | | 0.973-0,984 | [3] |
| Post-relapse follow-up → death | | 0.016-0.027 | [3] |

- - 1. Initial Ranges Curative Radiotherapy

Table 7: Initial ranges for transition probabilities associated with curative radiotherapy used in calibration process

| Variable |  | Range | Reference |
| --- | --- | --- | --- |
| Disease-free → disease-free | | 0.977-0.989 | [4] |
| Disease-free → locoregional relapse | | 0.003-0.005 | [4] |
| Disease-free → death | | 0.009-0.018 | [4] |
| Locoregional relapse → post-relapse follow-up | | 0.973-0,984 | [3] |
| Locoregional relapse → death | | 0.016-0.027 | [3] |
| Post-relapse follow-up → post-relapse follow-up | | 0.973-0,984 | [3] |
| Post-relapse follow-up → death | | 0.016-0.027 | [3] |

- - 1. Calibrated Values Curative Chemoradiotherapy

Table 8: Best fitting transition probabilities to data on outcomes associated with chemoradiotherapy achieved through calibration process

| Variable |  | Value | Reference |
| --- | --- | --- | --- |
| Disease-free → disease-free | | 0.977 | Calibration |
| Disease-free → locoregional relapse | | 0.090 |  |
| Disease-free → death | | 0.014 |  |
| Locoregional relapse → post-relapse follow-up | | 0.999 |  |
| Locoregional relapse → death | | 0.001 |  |
| Post-relapse follow-up → post-relapse follow-up | | 0.999 |  |
| Post-relapse follow-up → death | | 0.001 |  |

heitiitial values used ss were alibration process were om the 00000000000000000000000000000000000000000000000000000000000000000

- - 1. Curative Radiotherapy

Table 9: Best fitting transition probabilities to data on outcomes associated with radiotherapy achieved through calibration process

| Variable |  | Value | Reference |
| --- | --- | --- | --- |
| Disease-free → disease-free | | 0.946 | Calibration |
| Disease-free → locoregional relapse | | 0.032 |  |
| Disease-free → death | | 0.022 |  |
| Locoregional relapse → post-relapse follow-up | | 0.995 |  |
| Locoregional relapse → death | | 0.005 |  |
| Post-relapse follow-up → post-relapse follow-up | | 0.995 |  |
| Post-relapse follow-up → death | | 0.005 |  |

- - 1. Salvage Surgery

Table 10: Probability of undergoing salvage surgery after locoregional relapse

| Variable |  | Value | Reference |
| --- | --- | --- | --- |
| Proportion undergoing salvage surgery | | 0.730 | [3] |

For both curative groups it was assumed the same proportion would undergo salvage surgery after locoregional relapse, despite their being a higher probability that those undergoing radiotherapy alone would not be suitable for resection. This assumption was chose as there is no data on salvage rates in those receiving combination therapy versus radiotherapy alone, and given the very small number of patients in the latter group it was felt that this assumption would have limited impact on the overall results.

- - 1. Palliative Care

Table 11: Transition probabilities for those receiving palliative care

| Variable |  | Value | Reference |
| --- | --- | --- | --- |
| Palliative care → palliative care | | 0.962 | [5] |
| Palliative care → dead | | 0.038 | [5] |

Mortality for those receiving palliative care was based on a 5 year survival rate for those with advanced stage anal cancer at diagnosis of 10%, taken from Cancer Research UK [5].

1. Costs

All the cost inputs used in the model can be found in Table 14. A market force factor of 1.08 was applied to all unit costs to account for regional variation in prices [6].

- 1. Referral

Costs for referral by a GP were based on the assumption that patients would have on average two consultations before being referred to a specialist. This cost does not take into account any treatments which may have been prescribed during the first consultation and therefore represents a conservative estimate. GUM physicians were assumed to be more familiar with the signs of anal cancer and therefore costs were based on only an average of 1.5 consultations.

- 1. Staging

After referral, patients were assumed to have an MRI and a CT scan, followed by an excision biopsy. The latter was assumed to be delivered as an inpatient procedure based on the patterns observed in the HES dataset, with the rest carried out in the outpatient setting. A proportion of patients were also assumed to undergo node sampling using fine needle aspiration [1].

- 1. Primary Treatment
     1. Curative Chemoradiotherapy and Radiotherapy

Initial costs for patients in both groups were based on the assumption that all patients receive external beam irradiation totalling 45Gy in the first instance, delivered in 20 fractions [3, 7]. Costs for the chemoradiotherapy therapy group also included items to cover two infusions of 5-flouracil and single injection of mytomycin C. Booster doses were assumed to be 15Gy over 10 fractions.

Primary treatment costs were also assumed to contain discussion of a patient at a multidisciplinary team (MDT) meeting, an initial outpatient attendance with a clinical oncologist, radiotherapy planning and finally a follow-up outpatient attendance [8, 9]. The model takes no account of the costs of treating complications associated with either treatment regime. No currency code exist for colorectal MDTs therefore we assumed this to be the same cost as an initial consultant led outpatient attendance.

- - 1. Palliative Care

Costs for palliative radiotherapy and surgery included MDT discussion along with initial and follow-up outpatient attendances (colorectal surgery or clinical oncology depending on the intervention). Radiotherapy also involved planning and the delivery of five fractions of external beam radiation. Palliative surgery was assumed to involve minor anal procedures.

- 1. Follow-Up

The total cost of follow-up for each group (curative chemoradiotherapy, radiotherapy and palliative care) costs were determined by applying the proportions in each of the disease states in the various compartments of the Markov model to their associated costs, and then summing across all cycles.

- - 1. Curative Chemoradiotherapy and Radiotherapy

Follow-up regimes were assumed to be the same for both curative groups. As all patients were assumed to begin in the disease-free state, and therefore their time in this state could be traced, it was possible to apply costs only to those months where interventions would be expected as per the standard programme of follow-up for anal cancer patients. The months and interventions can be found in Table 12 [10]. This approach has probably led to some underestimation in the costs as some patients, for example those with higher grade primary tumours or evidence of lymph node involvement, would be more intensely followed-up due to their higher risk of relapse.

Table 12: Schedule of follow-up interventions for disease-free patients

| Month | Description | Reference |
| --- | --- | --- |
| 2 | Follow-up outpatient visit – surgery | [10] |
| 4 | Follow-up outpatient visit – surgery |  |
| 6 | Follow-up outpatient visit – surgery |  |
| 6 | MRI scan |  |
| 6 | CT scan |  |
| 8 | Follow-up outpatient visit – surgery |  |
| 10 | Follow-up outpatient visit – surgery |  |
| 12 | Follow-up outpatient visit – surgery |  |
| 15 | Follow-up outpatient visit – surgery |  |
| 18 | Follow-up outpatient visit – surgery |  |
| 21 | Follow-up outpatient visit – surgery |  |
| 24 | Follow-up outpatient visit – surgery |  |
| 30 | Follow-up outpatient visit – surgery |  |
| 36 | Follow-up outpatient visit – surgery |  |
| 42 | Follow-up outpatient visit – surgery |  |
| 48 | Follow-up outpatient visit – surgery |  |
| 54 | Follow-up outpatient visit – surgery |  |
| 60 | Follow-up outpatient visit – surgery |  |

The proportion of patients undergoing relapse in each cycle was multiplied by the proportion undergoing salvage to determine costs for this state. No additional costs were applied to those not undergoing salvage which again has most likely led to some underestimation of the true costs. Salvage surgery was assumed to include four outpatient consultations, an abdominoperineal resection (APR) followed by some form of plastic reconstruction (for costs used to cover these procedures see Table 14).

Markov models are memoryless and therefore it was not possible to apply the same approach that was used to estimate the follow-up costs for those who remained disease free for those undergoing follow-up post-relapse. For this group we calculated the total costs of the full follow-up programme for high-risk patients and then divided this by the total number of months over which it would be expected to take place (ten years, or 120 months) to give us an average monthly cost of follow-up of £65 [10]. This was then applied to the proportion of patients in this disease state. This approach has probably also led to some underestimation in the costs of follow-up for high-risk patients and consequently the overall total costs of treatment.

Table 13: Interventions comprising the ten years follow-up regime for high risk patients

| Volume | Description | Reference |
| --- | --- | --- |
| 16 | Follow-up outpatient visit – surgery | [10] |
| 6 | MRI scan |  |
| 3 | CT scan |  |

- - 1. Palliative Care

Monthly costs for those undergoing palliative care were calculated by averaging monthly costs from a study of a number of other cancer areas [11]. The study included data on breast, colon, lung, uterus, ovary, oesophagus and stomach cancer patients. The average monthly cost used in the model was £315.

- 1. Inflation

Treatment was assumed to begin at the start of 2010/11. In the scenario where inflation was taken into account, the level recorded in 2011/12 (1.028%) was applied to the total costs of the second twelve months of follow-up, and all subsequent twelve month periods [12].

Table 14: Unit costs used in model calculations

| Intervention | Currency code | Description | Tariff/Cost | Reference |
| --- | --- | --- | --- | --- |
| GP visit | n/a |  | £36 | [13] |
| Initial outpatient visit – genito-urinary | 360 | Genito-Urinary Medicine | £133 | [14] |
| MRI scan | RA07Z | Magnetic Resonance Imaging Scan, requiring extensive patient repositioning and/or more than one contrast agent | £357 | [15] |
| CT scan  FNA | RA13Z  CZ36Y | Computerised Tomography Scan, three areas with contrast  Excision/biopsy procedures 19 years and over | £151  £492 | [15] |
| Biopsy | CZ36Y | Excision/biopsy procedures 19 years and over | £1530 | [15] |
| Initial outpatient visit – surgery | 104 | Colorectal Surgery | £139 | [14] |
| Initial outpatient visit – clinical oncology | 800 | Clinical Oncology | £193 | [14] |
| Follow-up outpatient visit – surgery | 104 | Colorectal Surgery | £81 | [14] |
| Follow-up outpatient visit – clinical oncology | 800 | Clinical Oncology | £84 | [14] |
| Radiotherapy Planning | SC52Z | Preparation for complex conformal radiotherapy – With Technical Support | £1,309 | [15] |
| Mitomycin C | N/A | n/a | £44 | Expert discussion |
| PICC line insertion | CZ32Y | Fitting/insertion procedures 19 years and over | £751 | [15] |
| 5-FU | SB02Z | Procure Chemotherapy drugs for regimens in Band 2 | £305 | [15] |
|  | SB14Z | Deliver complex Chemotherapy, including prolonged infusional treatment at first attendance | £334 | [15] |
|  | SB15Z | Deliver subsequent elements of a Chemotherapy cycle | £294 | [15] |
| Radiotherapy delivery | SC23Z | Deliver a fraction of complex treatment on a megavoltage machine | £134 | [15] |
| Palliative surgery | FZ23Z | Minor Anal Procedures | £625 | [14] |
| Salvage surgery | FZ08A | Complex Large Intestine Procedures with Major CC | £7,788 | [14] |
| Plastic reconstruction | FZ21Z | Major Anal Procedures | £1,102 | [14] |
| Pre-treatment colostomy | FZ11B | Large Intestine - Major Procedures without Major CC | £2043 | [14] |

1. Sensitivity Analysis

Details of the alternative values used in the one-way sensitivity analysis can be found in the following sections.

- 1. Proportion receiving Curative Care

Table 15: Alternative values for the proportion of patients receiving curative care used in the sensitivity analysis

| Scenario |  | Value | Reference |
| --- | --- | --- | --- |
| Upper | | 0.970 | Expert discussion |
| Lower | | 0.870 |  |

- 1. Proportion receiving Curative Chemoradiotherapy

Table 16: Alternative values for the proportion of patients receiving curative chemoradiotherapy used in the sensitivity analysis

| Scenario |  | Value | Reference |
| --- | --- | --- | --- |
| Upper | | 0.980 | Expert discussion |
| Lower | | 0.880 |  |

- 1. Monthly Cost of Palliative Care

Table 17: Alternative values for the monthly costs of palliative care used in the sensitivity analysis

| Scenario |  | Value | Reference |
| --- | --- | --- | --- |
| Upper | | £611 | [11] |
| Lower | | £194 | [11] |

- 1. Proportion undergoing Salvage Surgery

Table 18: Alternative values for the probability that patients who experience locoregional relapse will undergo salvage surgery used in the sensitivity analysis

| Scenario |  | Value | Reference |
| --- | --- | --- | --- |
| Upper | | 0.83 | Expert discussion |
| Lower | | 0.63 |  |

- 1. Mode of Admission for Chemoradiotherapy and Radiotherapy

Table 19: Alternative costs based on mode of admission for chemotherapy and radiotherapy used in the sensitivity analysis

| Scenario/Parameter |  | Value | Reference |
| --- | --- | --- | --- |
| *Upper* | |  |  |
| Radiotherapy Planning | | £2,869 | [15] |
| PICC line insertion | | £2,068 |  |
| Procure 5-FU | | £339 |  |
| Deliver first 5-FU infusion | | £334 |  |
| Deliver subsequent 5-FU infusion | | £294 |  |
| Radiotherapy delivery | | £261 |  |
| *Lower* | |  |  |
| Radiotherapy Planning | | £729 | [15] |
| PICC line insertion | | £751 |  |
| Procure 5-FU | | £286 |  |
| Deliver first 5-FU infusion | | £302 |  |
| Deliver subsequent 5-FU infusion | | £206 |  |
| Radiotherapy delivery | | £111 |  |

- 1. Intensity of Follow-Up

Table 20: High intensity schedule of follow-up interventions for disease-free patients

| Month | Description | Reference |
| --- | --- | --- |
| 2 | Follow-up outpatient visit – surgery | [10] |
| 4 | Follow-up outpatient visit – surgery |  |
| 6 | Follow-up outpatient visit – surgery |  |
| 6 | MRI scan |  |
| 6 | CT scan |  |
| 8 | Follow-up outpatient visit – surgery |  |
| 10 | Follow-up outpatient visit – surgery |  |
| 12 | Follow-up outpatient visit – surgery |  |
| 14 | Follow-up outpatient visit – surgery |  |
| 16 | Follow-up outpatient visit – surgery |  |
| 18 | Follow-up outpatient visit – surgery |  |
| 18 | MRI scan |  |
| 18 | CT scan |  |
| 20 | Follow-up outpatient visit – surgery |  |
| 22 | Follow-up outpatient visit – surgery |  |
| 24 | Follow-up outpatient visit – surgery |  |
| 30 | Follow-up outpatient visit – surgery |  |
| 36 | Follow-up outpatient visit – surgery |  |
| 42 | Follow-up outpatient visit – surgery |  |
| 48 | Follow-up outpatient visit – surgery |  |
| 54 | Follow-up outpatient visit – surgery |  |
| 60 | Follow-up outpatient visit – surgery |  |

Table 21: High intensity schedule of interventions comprising the ten years follow-up regime for high risk patients

| Volume | Description | Reference |
| --- | --- | --- |
| 18 | Consultant led follow-up outpatient visit | [10] |
| 7 | Magnetic Resonance Imaging Scan, requiring extensive patient repositioning and/or more than one contrast agent |  |
| 4 | Computerised Tomography Scan, three areas with contrast |  |

Table 22: Low intensity schedule of follow-up interventions for disease-free patients

| Month | Description | Reference |
| --- | --- | --- |
| 2 | Outpatient visit | [10] |
| 4 | Outpatient visit |  |
| 6 | Outpatient visit |  |
| 8 | Outpatient visit |  |
| 10 | Outpatient visit |  |
| 12 | Outpatient visit |  |
| 18 | Outpatient visit |  |
| 24 | Outpatient visit |  |
| 30 | Outpatient visit |  |
| 36 | Outpatient visit |  |
| 42 | Outpatient visit |  |
| 48 | Outpatient visit |  |
| 54 | Outpatient visit |  |
| 60 | Outpatient visit |  |

Table 23: Low intensity schedule of interventions comprising the ten years follow-up regime for high risk patients

| Volume | Description | Reference |
| --- | --- | --- |
| 14 | Outpatient visit | [10] |
| 5 | MRI scan |  |
| 2 | CT scan |  |

- 1. Cost of Follow-Up Interventions

Table 24: Alternative values for costs of follow-up outpatient interventions used in the sensitivity analysis

| Scenario/Parameter |  | Value | Reference |
| --- | --- | --- | --- |
| *Upper* | |  |  |
| MRI scan | | £413 | [15] |
| CT scan | | £178 |  |
| *Lower* | |  |  |
| MRI scan | | £235 | [15] |
| CT scan | | £116 |  |

- 1. Cost of Staging and Primary Treatment

Table 25: Upper values for costs of staging and primary treatment interventions used in the sensitivity analysis

| Scenario/Parameter |  | Value | Reference |
| --- | --- | --- | --- |
| MRI scan | | £413 | [15] |
| CT scan | | £178 |  |
| Fine needle aspiration | | £616 |  |
| Biopsy | | £1,797 |  |
| Radiotherapy planning | | £1,309 |  |
| Mytomycin C | | £48 |  |
| PICC line insertion | | £877 |  |
| Procure 5-FU | | £360 |  |
| Deliver first 5-FU infusion | | £401 |  |
| Deliver subsequent 5-FU infusion | | £343 |  |
| Radiotherapy delivery | | £134 |  |

Table 26: Lower values for costs of staging and primary treatment interventions used in the sensitivity analysis

| Scenario/Parameter |  | Value | Reference |
| --- | --- | --- | --- |
| MRI scan | | £235 | [15] |
| CT scan | | £116 |  |
| Fine needle aspiration | | £269 |  |
| Biopsy | | £1,116 |  |
| Radiotherapy planning | | £674 |  |
| Mytomycin C | | £40 |  |
| PICC line insertion | | £414 |  |
| Procure 5-FU | | £187 |  |
| Deliver first 5-FU infusion | | £232 |  |
| Deliver subsequent 5-FU infusion | | £210 |  |
| Radiotherapy delivery | | £122 |  |

1. References

1. Branagan G: **Staging and management of inguinal nodes.** *Colorectal Dis* 2011, **13** (Suppl 1):29–32.

2. O’Dwyer S: **The management of anal cancers. UKACR Colorectal Study Day – NYCRIS.** 2011.

3. Renehan AG, Saunders MP, Schofield PF, O’Dwyer ST: **Patterns of local disease failure and outcome after salvage surgery in patients with anal cancer.** *Br J Surg* 2005, **92**:605–14.

4. Northover J, Glynne-Jones R, Sebag-Montefiore D, James R, Meadows H, Wan S, Jitlal M, Ledermann J: **Chemoradiation for the treatment of epidermoid anal cancer: 13-year follow-up of the first randomised UKCCCR Anal Cancer Trial (ACT I).** *Br J Cancer* 2010, **102**:1123–8.

5. **Statistics and outlook for cancer of the anus** [http://www.cancerresearchuk.org/cancer-help/type/anal-cancer/treatment/statistics-and-outlook-for-cancer-of-the-anus]

6. **Payment by Results in the NHS: A Simple Guide** [https://www.gov.uk/government/publications/simple-guide-to-payment-by-results]

7. Kronfli M, Glynne-Jones R: **Chemoradiotherapy in anal cancer.** *Colorectal Dis* 2011, **13** (Suppl 1):33–8.

8. Renehan AG, O’Dwyer ST: **Initial management through the anal cancer multidisciplinary team meeting.** *Colorectal Dis* 2011, **13** (Suppl 1):21–8.

9. Salmo E, Haboubi N: **Anal cancer: pathology, staging and evidence-based minimum data set.** *Colorectal Dis* 2011, **13** (Suppl 1):11–20.

10. Myint AS: **Follow up.** *Colorectal Dis* 2011, **13** (Suppl 1):39–43.

11. Guest JF, Ruiz FJ, Greener MJ, Trotman IF: **Palliative care treatment patterns and associated costs of healthcare resource use for specific advanced cancer patients in the UK.** *Eur J Cancer Care (Engl)* 2006, **15**:65–73.

12. **Consumer Price Indices (August Dataset)** [http://www.ons.gov.uk/ons/rel/cpi/consumer-price-indices/august-2012/cpi-time-series-data.html]

13. Curtis L: *Unit Costs of Health and Social Care*. PSSRU: Kent; 2011.

14. **2010-11 National Tariff** [http://data.gov.uk/dataset/payment-by-results-2010-11-national-tariff-information]

15. **2010-11 Reference Costs** [https://www.gov.uk/government/publications/2010-11-reference-costs-publication]
